# Supplementary material for: A-site cation influence on the conduction band of lead bromide perovskites
Source: Nat Commun. 2022 Jul 4;13:3839. doi: 10.1038/s41467-022-31416-y (PMC9253039; doi:10.1038/s41467-022-31416-y)
Supplement: Supplementary file 1 — Supplementary Information [file 41467_2022_31416_MOESM1_ESM.pdf]

Supplementary Information - A-site cation influence on the conduction band of lead bromide perovskites

Gabriel J. Man*[1]†, Chinnathambi Kamal[2,3,4], Aleksandr Kalinko[5], Dibya Phuyal[6], Joydev Acharya[7], Soham Mukherjee[1], Pabitra K. Nayak[7], Håkan Rensmo[1], Michael Odelius[2] and Sergei M. Butorin*[1]

Author addresses:

1. Condensed Matter Physics of Energy Materials, Division of X-ray Photon Science, Department of Physics and Astronomy, Uppsala University, Box 516, Uppsala 75121, Sweden
2. Department of Physics, Stockholm University, AlbaNova University Center, Stockholm 10691, Sweden
3. Theory and Simulations Laboratory, HRDS, Raja Ramanna Centre for Advanced Technology, Indore 452013, India
4. Homi Bhabha National Institute, Training School Complex, Anushakti Nagar, Mumbai 400094, India
5. Photon Science DESY, Notkestraße 85, Hamburg 22607, Germany
6. Division of Material and Nano Physics, Department of Applied Physics, KTH Royal Institute of Technology, Stockholm 10691, Sweden
7. Tata Institute of Fundamental Research, 36/P, Gopanpally Village, Serilingampally Mandal, Hyderabad 500046, India

* Correspondence and requests for materials should be addressed to G.J.M. (gman@alumni.princeton.edu) and S.M.B. (sergei.butorin@physics.uu.se)

† Present address: GJM Scientific Consulting, Fort Lee, New Jersey 07024, United States of America

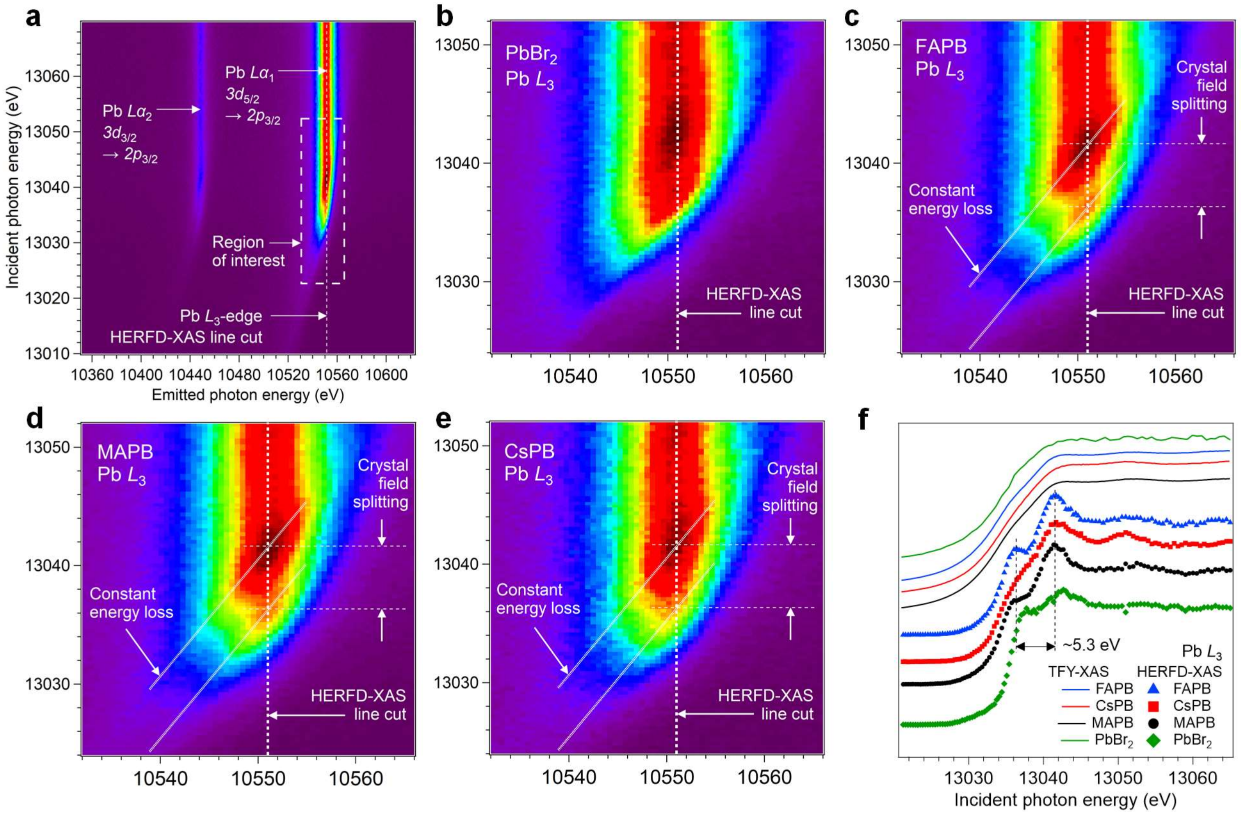

Supplementary Fig. 1. **Lead L_3 RXES maps and HERFD-XAS spectra of $PbBr_2$, FAPB, MAPB and CsPB.** The High Energy Resolution Fluorescence Detected X-ray Absorption Spectroscopy (HERFD-XAS) line cuts are visualized as dashed white vertical lines in all maps. **a** Representative Resonant X-ray Emission Spectroscopy (RXES) full map recorded from single crystal MAPB. Two core-to-core transitions ($L\alpha_{1,2}$) were measured with the energy window provided by the spectrometer. The near-edge features are contained within the region of interest. **b-e** Regions of interest of $PbBr_2$, FAPB, MAPB and CsPB. Diagonal and horizontal dashed white lines are guides to the eye, highlighting constant energy loss features related to octahedral crystal field splitting in the lead *6d* shell. The x- and y-axis labels for the regions are the same as the full map. **f** Comparison of Total Fluorescence Yield X-ray Absorption Spectroscopy (TFY-XAS, lines) and HERFD-XAS (symbols) spectra of $PbBr_2$ (green), FAPB (blue), MAPB (black) and CsPB (red). The magnitude of the Pb *6d* octahedral crystal field splitting (~5.3 eV) is estimated from the FAPB spectrum, which shows the sharpest features of the three APB compounds.

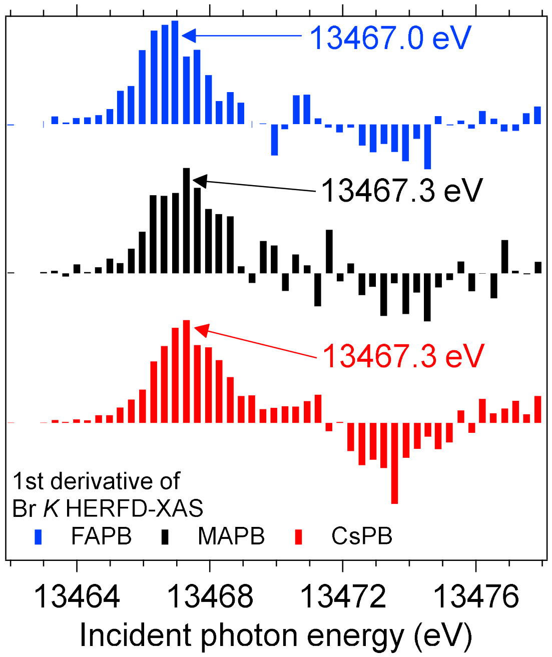

Supplementary Fig. 2. **First derivative of Br *K* HERFD-XAS spectra of FAPB, MAPB and CsPB.** The original Bromine *K*-edge High Energy Resolution Fluorescence Detected X-ray Absorption Spectroscopy (Br *K* HERFD-XAS) spectra are displayed in Fig. 2b. The photon energy associated with the inflection point in the absorption onset is displayed in the plot.

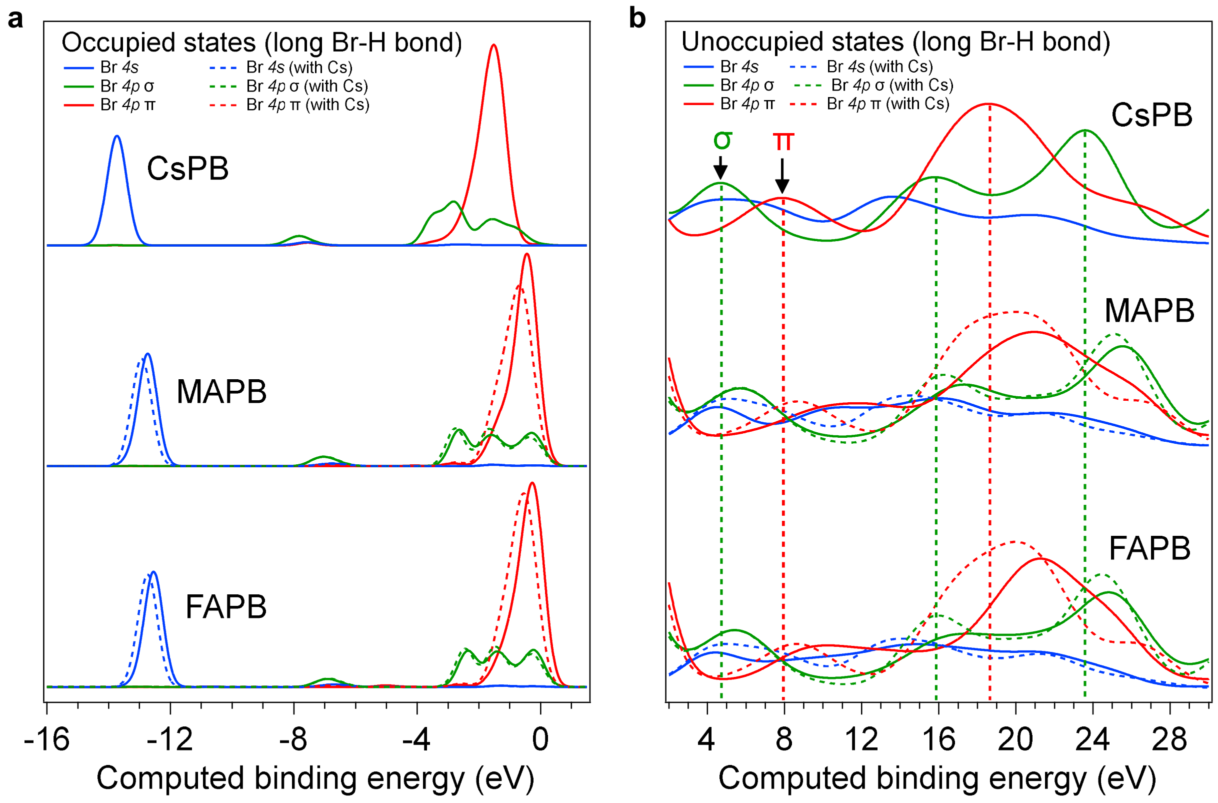

Supplementary Fig. 3. **Calculated ground-state bromine *4s*, *4p* σ and *4p* π projected density of states of CsPB and weakly hydrogen-bonded FAPB and MAPB**. **a** Occupied states are enumerated with negative binding energies and are Gaussian-broadened by 0.3 eV. The top of the valence band is aligned to 0 eV binding energy. **b** Unoccupied states are Gaussian-broadened by 1.0 eV to emphasize the main features of interest. The two π components have been averaged together to yield one Br *4p* π distribution. Projected density of states (PDOS) corresponding to weakly hydrogen-bonded bromine with organic molecules are shown. Bromine PDOS associated with cesium-substituted MAPB/FAPB are shown with dashed lines. The vertical dashed lines are guides to the eye and mark CsPB features of interest that can be compared to similar features for original and cesium-substituted MAPB and FAPB.

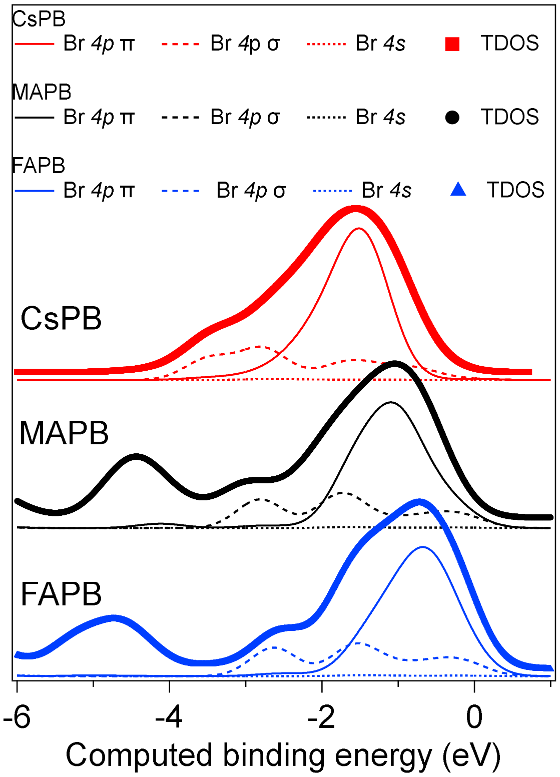

Supplementary Fig. 4. **Calculated ground-state bromine *4s*, *4p* σ and *4p* π projected density of states and total density of states of CsPB, FAPB and MAPB.** Occupied states are Gaussian-broadened by 0.3 eV. The top of the valence band is aligned to 0 eV binding energy. Projected density of states (PDOS) corresponding to strongly hydrogen-bonded bromine with organic molecules are shown. Total density of states (TDOS) are shown with symbols while the PDOS are shown with solid and dashed lines. The MAPB and FAPB TDOS features between -3.5 to -6 eV originate from the organic A-cations. The calculations were performed with CP2K.

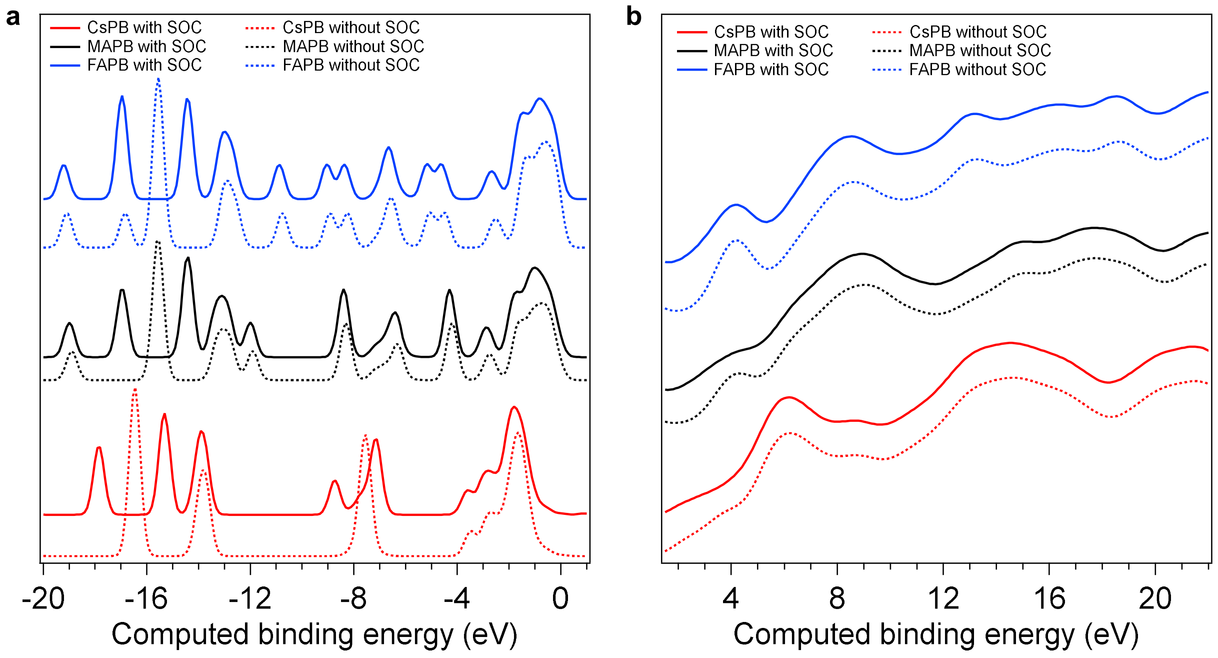

Supplementary Fig. 5. **Calculated total electronic density of states of CsPB, MAPB, and FAPB with and without spin-orbit coupling effects.** **a** Occupied states are enumerated with negative binding energies and are Gaussian-broadened by 0.3 eV. **b** Unoccupied states are Gaussian-broadened by 1.0 eV to emphasize the main features of interest. Calculated total density of states (TDOS) without spin-orbit coupling (SOC) effects are shown with dashed lines, and TDOS with SOC effects are shown with solid lines. The calculations were performed with Quantum Espresso.

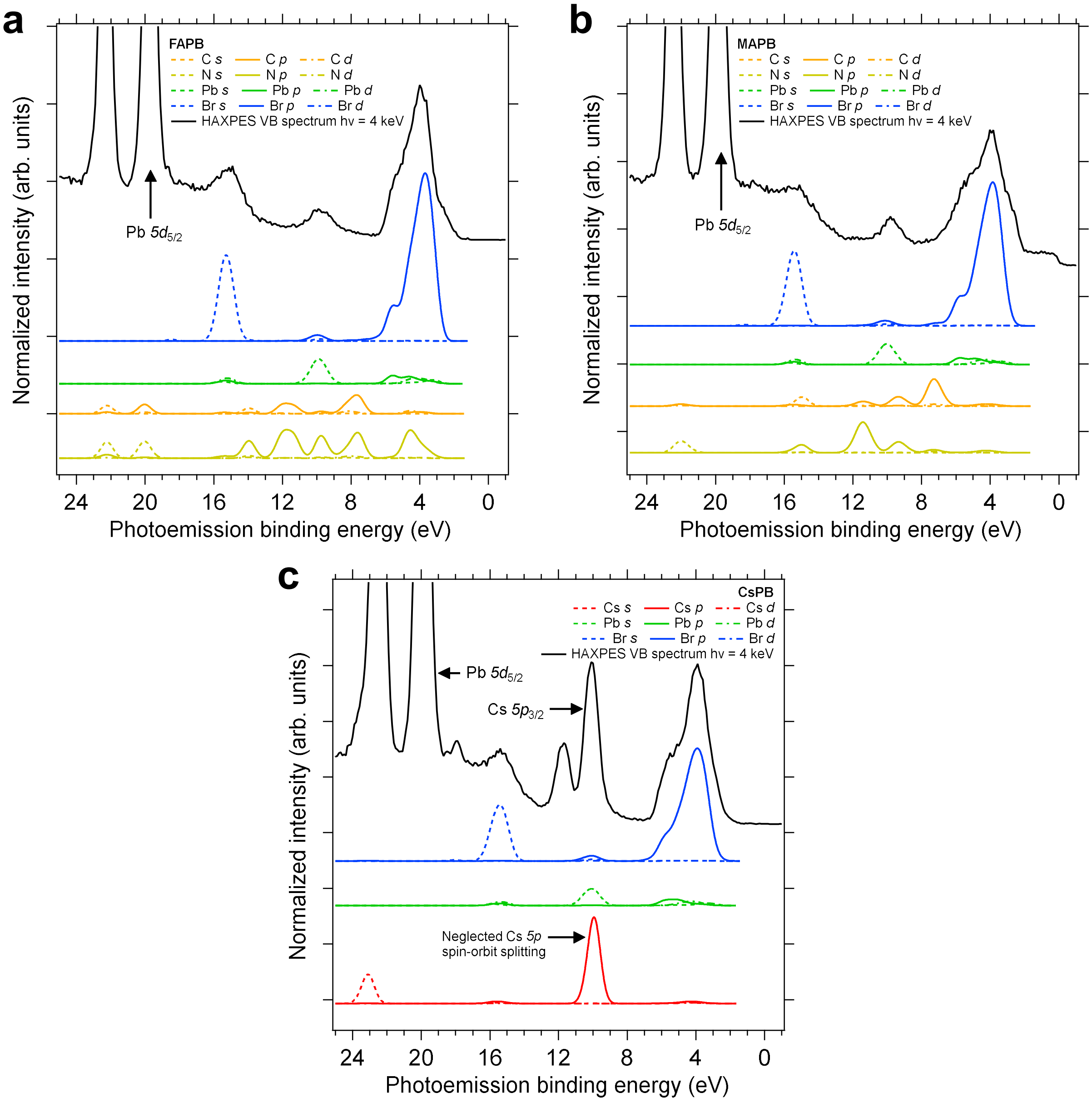

Supplementary Fig. 6. **Experimental valence band photoelectron spectra compared to calculated ground-state element- and orbital-projected DOS.** **a** FAPB. **b** MAPB. **c** CsPB. Occupied states, for both experiment and calculations, are enumerated with positive binding energies, consistent with the Photoelectron Spectroscopy (PES) convention. The calculated density of states (DOS) were Gaussian-broadened by 0.3 eV to match the energy resolution of the hard X-ray PES (HAXPES) measurements. The MAPB valence band spectrum shows a Fermi step which likely originates from silver epoxy (see Supplementary Note 7).

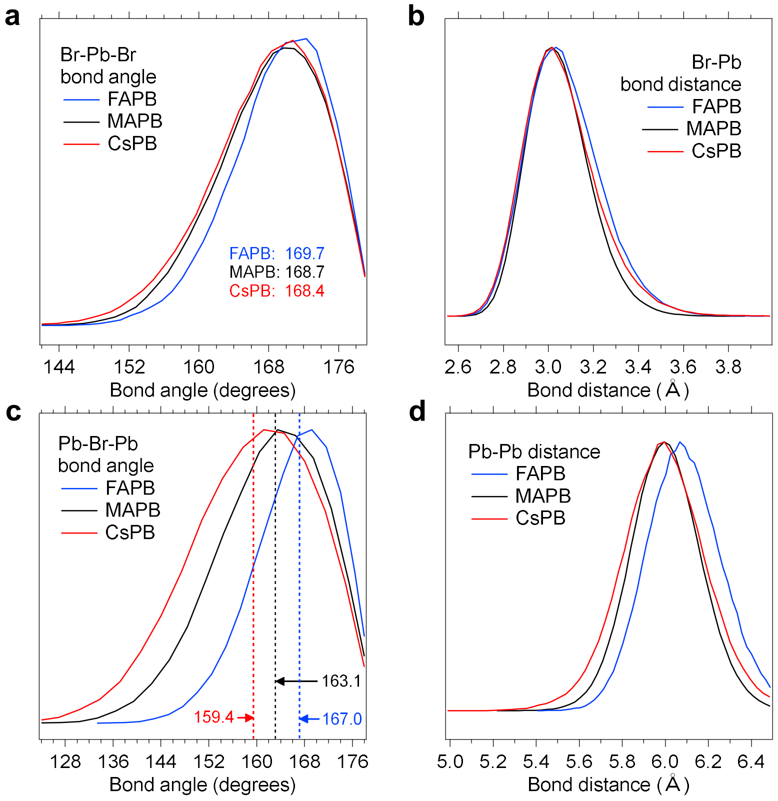

Supplementary Fig. 7. **Distributions of bond angles and bond distances derived from AIMD simulations.** Bond angles and bond distances for FAPB, MAPB and CsPB are based on snapshots outputted from ab initio molecular dynamics (AIMD) simulations. **a** Distributions of bromide-lead-bromide (Br-Pb-Br) bond angle. The Br-Pb-Br bond angles displayed in the panel are calculated center-of-gravity values. **b** Distributions of bromide-lead (Br-Pb) bond distance. **c** Distributions of lead-bromide-lead (Pb-Br-Pb) bond angle. The Pb-Br-Pb bond angles displayed in the panel are calculated center-of-gravity values. **d** Distributions of lead-lead distance.

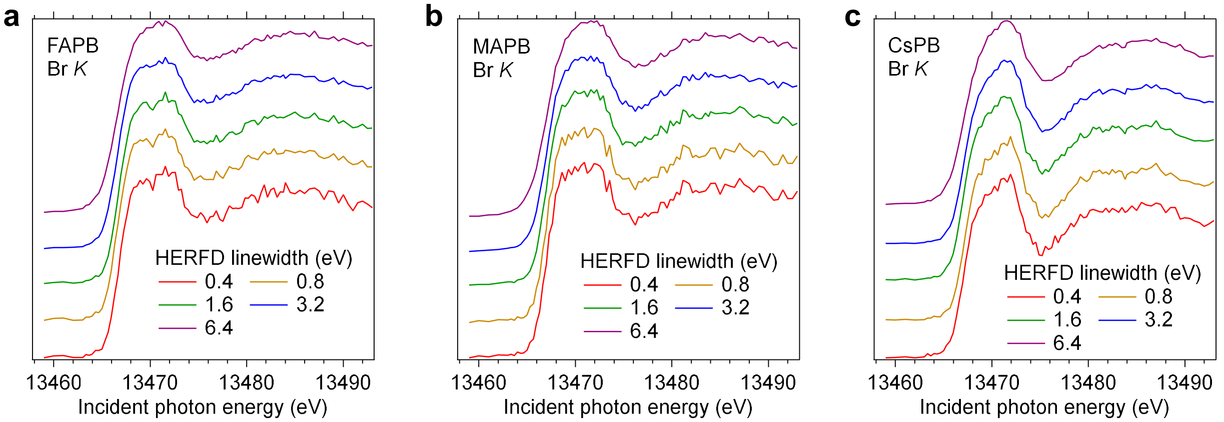

Supplementary Fig. 8. **Br *K* HERFD-XAS spectra displayed with a range of linewidths.** **a** FAPB. **b** MAPB. **c** CsPB. The linewidth refers to the energetic width of the slice on the emitted energy axis. The cut was done through the Resonant X-ray Emission Spectroscopy (RXES) maximum.

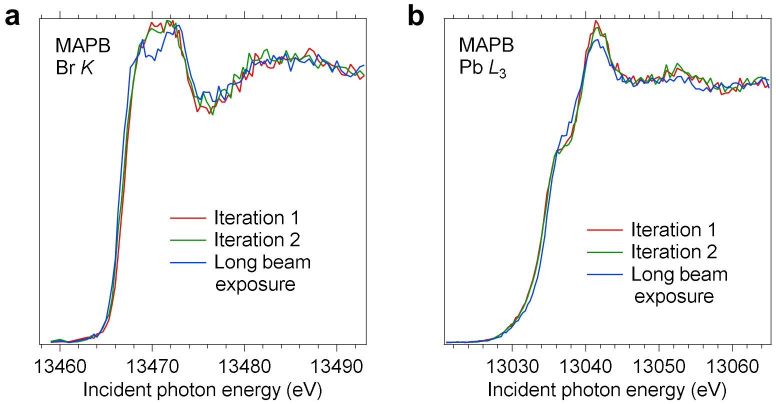

Supplementary Fig. 9. **Beam damage checks with Br *K* and Pb L_3 HERFD-XAS measurements**. Repeated measurements of the same type were performed at the same spot of the single crystal sample. Each measurement type was performed at a fresh spot. **a** Bromine *K*-edge High Energy Resolution Fluorescence Detected X-ray Absorption Spectroscopy (Br *K* HERFD-XAS) measurements recorded from MAPB. The spectra associated with iterations 1, 2 and 13 (long beam exposure) are shown. **b** Lead L_3-edge HERFD-XAS measurements recorded from MAPB. The spectra associated with iterations 1, 2 and 13 (long beam exposure) are shown. See Methods for further details.

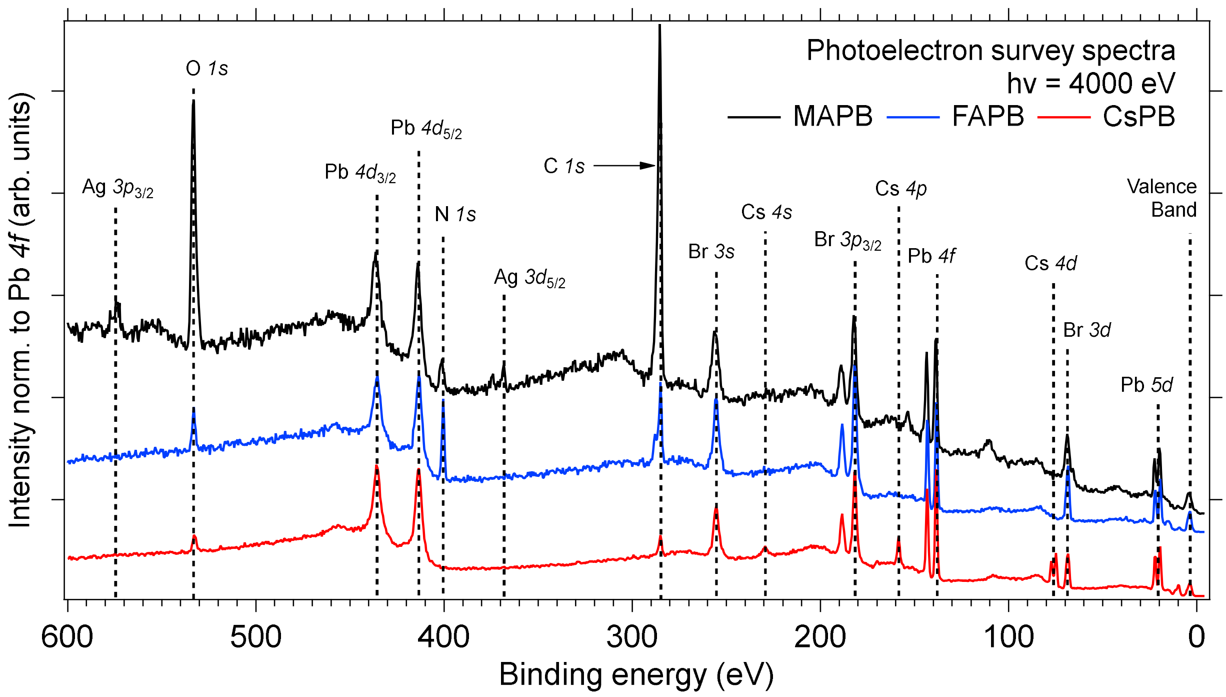

Supplementary Fig. 10. **Photoelectron survey/overview spectra recorded from single crystals of FAPB, MAPB and CsPB.** The spectra were recorded from uncleaved single crystal surfaces.

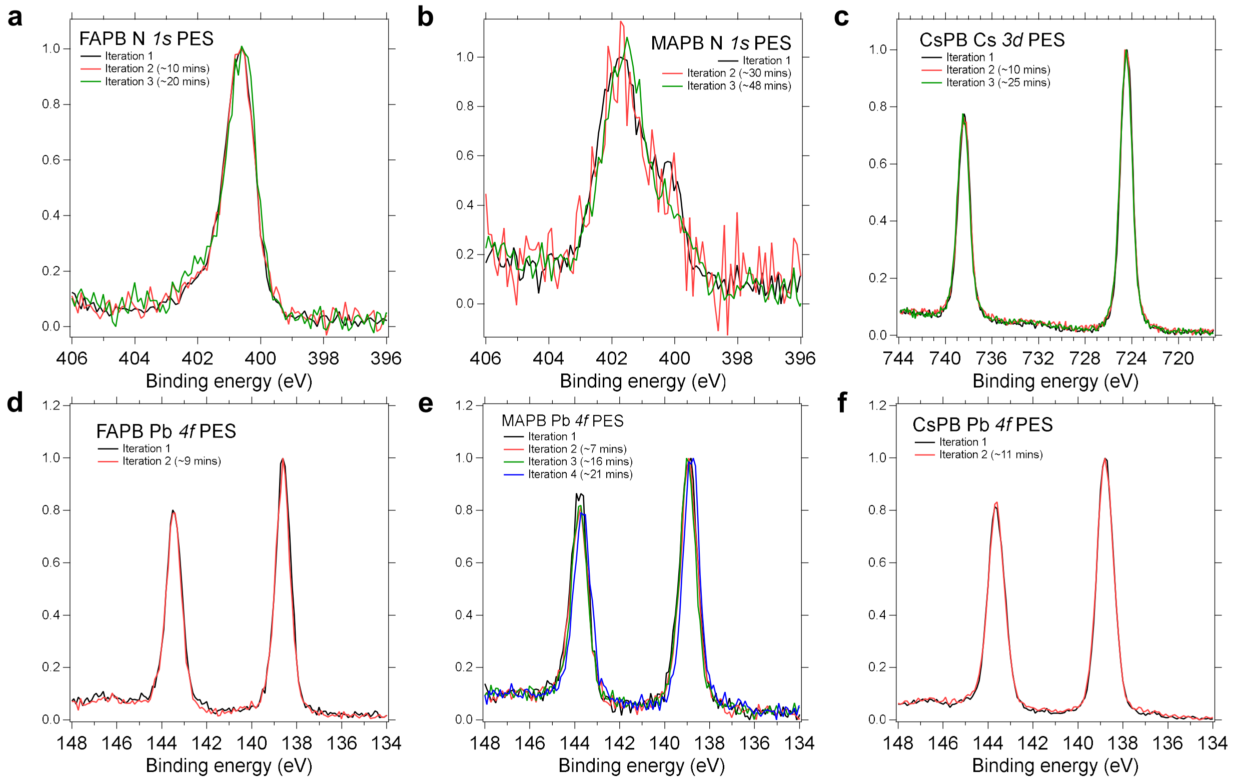

Supplementary Fig. 11. **Beam damage checks with photoelectron core level measurements.** Nitrogen *1s* (FAPB, MAPB) or cesium *3d* (CsPB) and lead *4f* (all) core level photoelectron spectra recorded from **a,d** FAPB, **b,e** MAPB and **c,f** CsPB. For each compound, all core level spectra were recorded from the same spot of the single crystal sample. The time(s) associated with iteration(s) ≥ 2 represents the elapsed time since the first iteration was recorded. During this time, the same spot was continuously exposed to the X-ray beam. The y-axis label for all plots is normalized intensity in arbitrary units.

Supplementary Table 1. **Ground-state σ-π splitting derived from calculated Br *4p* σ and π projected density of states in the unoccupied states.**

		Ground-state σ-π energy splitting	
Compound	With/without cesium substitution	Short Br-H bond distance	Long Br-H bond distance
FAPB	Original	7.1	5.2
	With cesium	3.0	3.2
MAPB	Original	4.2	5.0
	With cesium	3.4	3.0
CsPB	Original	3.2	
	With cesium		

Supplementary Table 2. **Summary of crystallographic parameters of FAPB, MAPB and CsPB.**

	Experiment					Literature					Ref.
	Space Group	a, b, c (Å)	α=β =γ (°)	Volume (Å^3)	Z	Space Group	a, b, c (Å)	α=β =γ (°)	Volume (Å^3)	Z	
CsPB	Pnma	a = 8.2226(6) b = 11.7479(9) c = 8.2635(6)	90	798.24 (10)	4	Pnma	a = 8.2440(6) b = 11.7351(11) c = 8.1982(8)	90	793.13 (12)	4	[1]
MAPB	Pm3m	5.928755	90	208.40	1	Pm3m	5.901(1)	90	205.49	1	[2]
FAPB	$Pm\bar{3}m$	a = 6.009(8)	90	217.06	1	$Pm\bar{3}m$	5.98618 (2)	90	214.511 (3)	1	[3]

Supplementary Table 3. **Photoionization cross-sections at hv = 4000 eV from the literature.** Polarization effects are not accounted for here.

Core level	Photo-ionization cross-section (σ) at hv = 4 keV in 10^{-21} cm^2 [4,5]	Relative photo-ionization cross-section, taking σ(Br *4p*$_{3/2}$) as unity
H *1s*	0.21e-3	
C *2p*$_{1/2}$	0.42e-3	
N *2p*$_{3/2}$	0.25e-2	
Br *4s*	0.360	1.2
Br *4p*$_{3/2}$	0.294	1.0
Cs *5p*$_{3/2}$	0.578	2.0
Cs *6s*	0.262e-1	~0.1
Pb *6s*	0.184	0.6
Pb *6p*$_{1/2}$	0.123	0.4

Supplementary Note 1. **Analysis of the Pb L_3 RXES full map and regions of interest, and HERFD-XAS spectra.**

A representative Pb L_3 RXES map, recorded from single crystal MAPB, is displayed in Supplementary Fig. 1a. Resonant and off-resonant X-ray emission was measured from two $3d \rightarrow 2p_{3/2}$ transitions ($L\alpha_{1,2}$). The ROI highlights the near-edge features of interest. The Pb L_3 ROIs for $PbBr_2$, FAPB, MAPB and CsPB (Supplementary Fig. 1b-e) show noticeable differences for incident photon energies below ~13042 eV. We observe diagonal constant energy loss features for all of the compounds, where the band of loss features is energetically narrower for $PbBr_2$ and wider for the APB compounds, and the edges of this band are sharper for FAPB versus CsPB. The constant energy loss features for the APB compounds intersect the HERFD-XAS line cut at ~13036 and ~13041 eV, giving rise to two prominent features in the Pb L_3 HERFD-XAS spectrum. These HERFD-XAS features have previously been assigned to excitations from Pb $2p_{3/2}$ to Pb *6d* states hybridized with Br states, where the energy split between the two features originates from octahedral crystal/ligand field splitting [6].

The improvement in intrinsic energy resolution yielded by Pb L_3 HERFD-XAS (~2.5 eV), as compared to TFY-XAS (~6.1 eV), is substantial and crucial for detecting differences in spectral features. Supplementary Fig. 1f visualizes this point. This demonstrates the sensitivity of HERFD-XAS to chemical state and/or crystal structure changes as demonstrated by past Pb $L_{3,1}$ HERFD-XAS studies of lead(II) compounds with different lead-ligand coordination and local structure[7].

Supplementary Note 2. **Comparison between experimental and calculated Br *K* XAS spectra of FAPB, MAPB and CsPB.**

In all cases, Fig. 2c-e, we observe that the heights of the main-edge profile (up to ~13476 eV) relative to the post- edge region (above ~13476 eV) appear smaller in the calculated spectra than in experiment. We also see in the calculations that the post-edge in each system, above ~13476 eV, is built up from two bands with σ-symmetry and a central π- symmetry band, but that is not resolved in the experiment. The small π-bonding contributions at similar excitation energies to the σ-bonding contributions in the main-edge, from ~13464 to ~13469 eV, may appear because the Pb-Br-Pb is bent.

We examine the computed peak positions of the σ main-edge feature, the main constituent of the absorption onset, and find a trend where the peak position increases, going from FAPB (13468.2 eV) to MAPB (13468.3 eV) to CsPB (13468.4 eV). The A-cation-influenced trend in the σ-π splitting, one of the key findings in our work, is unaffected by the Br *K* absorption onset (where our calculations show 0.1 eV relative changes) as the splitting spans several eV of the conduction band region. Our XAS calculations do correctly reproduce the absorption onset trend (increases in this order: CsPB → MAPB → FAPB). While the relative differences between MAPB and FAPB (experiment: 0.5 eV, calculation: 0.1 eV) were not fully captured, the relative differences between CsPB and MAPB (experiment: 0.1 eV, calculation: 0.1 eV) were fully captured. Overall, the quantitative σ-π splitting trend and the qualitative absorption onset trend hold.

The transition potential DFT methodology has been demonstrated to give useful support to the analysis of experimental data in numerous applications: (i) different element *K*-edge XAS and (ii) molecular and condensed phases. It is an approximate method, but has the advantage that it often gives semi-

quantitative results combined with an excellent scaling to large systems. Ekimova et al. [8] used a similar methodology to investigate hydrogen-bonded solutes in an environment of 63 water molecules, and Hou et al. [9] used a similar methodology to investigate nitrogen-doped graphene. Dalpian et al. [10] have shown that it is essential to use a large supercell for accurately modeling the ground-state crystal structures of HaPs; this necessitates the use of methods with excellent scaling characteristics.

Supplementary Note 3. **Potential DFT-related limitations with the calculation of the conduction band electronic structure.**

The common exchange-correlation functionals used in DFT are known to yield underestimated band-gaps. However, the occupied and unoccupied parts of the computed DOS are generally in good agreement with experimental photoelectron spectra, stretched by a few percent [11]. Within the conduction band region, systematic DFT underestimation of a few percent or ~ 0.1 eV is not expected to affect the σ-π splitting trend as the magnitude of the splitting is ~ 4 eV.

DFT, using the same exchange-correlation functional as what we have used (Perdew-Burke-Ernzerhof (PBE) generalized gradient approximation (GGA)), has been demonstrated to be reasonably accurate at modeling the conduction band electronic structure of MAPI, a closely related compound [12]. While there is overall agreement between the calculated conduction band dispersions and the measured bands (obtained via angle-resolved inverse photoelectron spectroscopy) over a ~3 eV region of the conduction band, the agreement is not perfect at the 0.1 eV order-of-magnitude energy scale. We also notice that the good performance of the PBE approximation for lead-based halide perovskites is related to a favorable error cancellation (e.g. Mosconi et al. [13], Das et al. [14]). It has been reported in the literature that the performance of standard GGA is good for a number of response properties (e.g. Drisdell et al. [6]).

Supplementary Note 4. **Analysis of Br *K* XES spectra.**

Bromine *K* XES provides a complementary bromine *p*-state-selective measurement of the occupied states to PES (but with significant (Br *1s* related) core hole lifetime and instrumental broadenings)[15]. To investigate the profile of the Br *4p* PDOS near the VBM (~13462 to ~13472 eV), the VtC main line in the XES spectra of FAPB, MAPB and CsPB was fitted with a Voigt peak. The range of the fit covers 13443 – 13481 eV. The fitted Voigt FWHM parameters are comparable (4.1-4.2 eV).

From Fig. 4 (center inset), we observe that the relative VtC intensity of FAPB is higher than MAPB/CsPB given normalized $K\beta_{1,3}$ intensities. This shows the Br *4p* electron occupancy is higher for FAPB and suggests the Br-Pb bond for FAPB is more ionic relative to MAPB/CsPB. Higher ionicity (or alternatively, lower covalency) means less Br *4p* electron sharing and hence higher orbital occupancy. To verify this finding, we examine the $K\beta_1$ and VtC transition energies. From an electrostatic standpoint, given the same formal oxidation state, the Br $K\beta_1$ transition energy is expected to be lower for a more ionic Br^- compound due to better shielding of the nucleus by the higher valence electron density. This leads to a lower effective nuclear charge and consequently to a lower excitation energy for a core electron. X-ray emission spectroscopy studies of I^--containing compounds show this trend with I $L\gamma$ emission[16]. Sulfur $K\alpha_1$ emission from sulfur-containing compounds shows a progression towards lower emission energies going from 6^+ to 2^- [17]. The Voigt peak-fitted Br $K\beta_1$ peak positions, or $3p_{3/2}\rightarrow 1s$ transition energies, are

13284.4 eV (FAPB), 13284.6 eV (MAPB) and 13284.7 eV (CsPB). The Voigt peak-fitted Br VtC maxima energies are 13462.1 eV (FAPB), 13462.4 eV (MAPB) and 13462.4 eV (CsPB). The uncertainties in the $K\beta_1$ and VtC fits are ± 4 and ± 23 meV, respectively. While the energy offset between the Br $3p_{3/2}$ and *4p* levels is essentially the same for all three compounds (~177.7 eV), the energy levels referenced to the Br *1s* level are different, revealing chemical shifts or differences in Br-Pb bond ionicity in spite of the same formal bromine oxidation state. The $K\beta_1$ transition energy shows an increasing trend of FAPB→MAPB→CsPB, with a FAPB-to-CsPB difference of 0.3 eV and MAPB-to-CsPB difference of 0.1 eV. The chemical shift, measured with ^{207}Pb NMR shows the same room-temperature FAPB→MAPB→CsPB trend with a FAPB-to-CsPB difference of 253 ppm and a MAPB-to-CsPB difference of 103 ppm[18]. Our Br *K* XAS investigation shows the same trend, with an increasing absorption onset trend of FAPB→MAPB→CsPB, indicating that the chemical shift applies to all occupied and unoccupied energy levels. The XAS and ground-state σ-π splittings both show a decreasing trend of FAPB→MAPB→CsPB, hence we find a correlation between A-cation and Br-Pb sublattice electronic coupling strength and Br-Pb bond ionicity. The Br-Pb bond ionicity trend is the inverse of the $K\beta_1$ transition energy trend and increases in this order: CsPB→MAPB→FAPB. Using frequency-dependent dielectric measurements, others have reported a trend where the Br-Pb bond in CsPB is more ionic relative to MAPB, contrary to our finding[19–21]. Experimental non-idealities such as electrical contacts may influence the dielectric measurements.

We notice however that the differences in Br-Pb bond ionicity are not captured in any charge analysis of the ground-state DFT calculations, despite the good agreement of these models in the analysis of the unoccupied states.

Supplementary Note 5. **Modeling valence band photoelectron spectra with calculated ground-state DOS.**

We utilize PES in its hard X-ray form, HAXPES, both to enhance the bulk sensitivity of the measurement and to emphasize the lead and bromide spectral contributions. Given the 4000 eV excitation used, the photo-ionization cross-sections of the Pb and Br states are 2-3 orders of magnitude higher than the cross-sections of carbon, hydrogen and nitrogen (Supplementary Table 3). The relative photoionization cross-sections for Pb *6s* (~0.6), Pb *6p* (~0.4) and Br *4p* (1.0) states at hv = 4 keV are comparable. Ground-state element- and orbital-projected DOS calculations, derived from the same underlying AIMD simulations used to calculate the Br *K* XAS spectra (Fig. 2c-e) and Br PDOS (Fig. 3) and Gaussian-broadened by 0.3 eV to match the experimental energy resolution, are compared to HAXPES valence band spectra in Supplementary Fig. 6. A Fermi edge/step feature is visible in the valence band spectrum of MAPB; the origin of the metallic states is likely the silver contamination, as mentioned in Supplementary Note 7. We note two observations from the comparison. First, most of the features in the valence band spectra of FAPB, MAPB and CsPB, extending from the Fermi energy to the Pb *5d* shallow core level can be approximately accounted for with the lead and bromine PDOS, except for the Cs *5p* semi-core level in the case of CsPB. This is consistent with the relatively low photo-ionization cross-sections of C *2p*, N *2p* and Cs *6s* states at hv = 4 keV. Second, the photoelectron spectra of FAPB (Supplementary Fig. 6a) and CsPB (Supplementary Fig. 6c), which are the endmembers of our set in terms of A-cation electronic coupling strength, show differences in the VBM DOS. This observation indicates that the A-cation replacement modifies the degree of Pb *6s* and/or *6p* hybridization with the Br

4p states, which may be relevant for optoelectronic functionality. Lead *6p* states predominate in the conduction band and the probabilities of visible optical absorption/radiation events, relevant for solar cells and LED's, may increase (due to $s \leftrightarrow p$ dipole selection rules for optical transitions) as the Pb *6s* contribution grows at the VBM. The degree of Rashba spin-splitting at the VBM, if present, may be modulated by changes in Pb *6p* contributions to the VBM as such states exhibit spin-orbit coupling[22].

Supplementary Note 6**. Connection between A-cation electronic coupling strength and crystal structure.**

We examine the crystal structure for manifestation(s) of A-cation electronic coupling, in terms of correlation(s) with the σ-π splitting, chemical shift, etc. The crystallographic parameters obtained from XRD measurements are summarized in Supplementary Table 2; these are comparable to literature values and show that the structural phases of CsPB, MAPB and FAPB are orthorhombic, cubic and cubic at ambient conditions (room temperature, 1 atm pressure).

The Pb L_3 HERFD-XAS spectra for the three APB compounds and $PbBr_2$ are shown in Supplementary Fig. 1f. We observe two prominent features in all of the spectra: a rising-edge feature at ~13036 eV and a main-edge feature at ~13041 eV. Drisdell et al. [6] have assigned the origin of these features to hybridized Br-Pb *6d* crystal field splitting. Between the three APB compounds, the sharpness of the two features has an apparent dependence on the type of A-cation and reveals differences in unit cell symmetry. The sharpness of the crystal field splitting features qualitatively increases in this order: CsPB→MAPB→FAPB. This indicates that the unit cell of FAPB shows the highest cubicity. The lower symmetry of $PbBr_2$ and CsPB leads to an additional broadening and smearing of the spectral structures compared to MAPB/FAPB.

We inspect several structural descriptors: (a) Br-Pb-Br bond angle, (b) Br-Pb bond distance, (c) Pb-Br-Pb bond angle and (d) Pb-Pb distance, obtained from AIMD simulations for correlation(s). The corresponding plots are displayed in Supplementary Fig. 7. Since the bond angle distributions are asymmetric, we use a center-of-gravity analysis to quantify the mean values. The Br-Pb-Br bond angle shows a systematic trend towards higher bond angle (CsPB→MAPB→FAPB) though the differences are small (mean bond angle for FAPB is ~0.8% greater than for CsPB) and the distributions of Br-Pb bond distance are comparable for the three APB compounds. This suggests the internal structure of the $PbBr_6$ octahedral unit is weakly affected by A-cation electronic coupling, though the effect on the electronic structure could still be substantial. On the other hand, the Pb-Br-Pb bond angle distribution shows a larger difference (i.e. the mean angle for FAPB is ~4.5% larger than the angle for CsPB) and a systematic CsPB→MAPB→FAPB trend towards higher angle. A larger Pb-Br-Pb bond angle signifies higher cubicity and unit cell symmetry, which is consistent with the Pb L_3 HERFD-XAS feature sharpness trend. The Pb-Br-Pb bond angle quantifies the degree of cooperative tilting of the $PbBr_6$ octahedra. The larger Pb-Pb distance for FAPB is consistent with the larger Pb-Br-Pb bond angle. From a purely geometrical standpoint, the Pb-Br-Pb bond angle is affected the most by the A-cation. We observe a positive correlation between the Pb-Br-Pb tilt angle and the XAS σ-π splitting, but are unable to validate the tilt as the mechanism responsible for the σ-π splitting using individual configurations. Hence, we deduce that the tilt angle and the σ-π splitting are not mechanistically related but both are consequences of the A-cation electronic coupling strength.

We examine the GTF, a structural descriptor which is applicable to all ABX_3 perovskites (where X = halide, oxide, fluoride, etc.), for a complementary view of the crystal structure[23]. The GTF was introduced nearly a century ago and is familiar to the oxide/fluoride/halide/etc. perovskite communities[23,24]. The GTF for APB compounds is defined as $t = \frac{r_{\mathrm{A}}+r_{\mathrm{Br}}}{\sqrt{2}(r_{\mathrm{Pb}}+r_{\mathrm{Br}})}$, where r represents the ionic radii of the A-cation, bromide anion or lead cation. Using r_{Pb} = 1.33 Å, r_{Br} = 1.82 Å and r_{Cs} = 1.81 Å from Shannon's database and reported computed values of $r_{\mathrm{MA+}}$ = 2.70 Å and $r_{\mathrm{FA+}}$ = 2.79 Å, we estimate GTF's of 1.03, 1.01 and 0.81 for FAPB, MAPB and CsPB, respectively[25,26]. Similar GTF's have been reported[27]. The trend of increasing GTF for CsPB(0.81)→MAPB(1.01)→FAPB(1.03) matches the trend in Pb L_3 HERFD-XAS sharpness of spectral structures related to the crystal field splitting. Our earlier analysis of the AIMD-derived structural descriptors showed the Pb-Br-Pb bond angle to vary the most between APB compounds, hence we deduce that the main factor which accounts for the different GTF's is the degree of cooperative octahedral tilting as quantified by the Pb-Br-Pb bond angle. From a structural standpoint, the GTF could be viewed as an approximate measure of the degree of octahedral tilting in APB compounds.

The relative GTF ratios for MAPB : FAPB and CsPB : FAPB are 0.98 and 0.79, respectively, nearly matching the relative main-edge width MAPB : FAPB ratios of 0.96 (experiment) and 0.95 (calculated) and CsPB : FAPB ratios of 0.83 (experiment) and 0.83 (calculated). Experiment refers to the relative Br *K* XAS main-edge width and calculated refers to the σ-π splitting derived from the calculated Br *K* XAS spectra. A positive and potentially linear correlation exists between relative GTF and relative σ-π splitting. Since we have found no evidence for a mechanistic relationship between the tilt angle (represented by the GTF) and the σ-π splitting (which we found to be affected by the A-cation electronic coupling strength), the apparent linear correlation between the σ-π splitting and GTF implies that the underlying mechanism which is responsible for both is the strength of electronic coupling between the A-cation and the bromide-lead sublattice.

Supplementary Note 7. **Analysis of HAXPES core level spectra and potential beam damage effects.**

The survey spectra were checked to ensure the expected elements are present (Supplementary Fig. 10). Since the crystals were uncleaved, C *1s* and O *1s* signals originating from surface contamination are observed. In addition, some unexpected silver contamination, likely originating from the silver epoxy used to bond the crystals to the sample plate, was observed from the surface region of MAPB. In general, all of the expected elements (e.g. bromine, lead, etc.) are present.

To assess the chemical integrity of the X-ray-irradiated surfaces, sequential measurements of core level spectra (Pb *4f* for the bromide-lead sub-lattice, Cs *3d* or N *1s* for the A-cation) were monitored for changes (Supplementary Fig. 11). For each sample, all core level spectra were recorded from the same spot on the crystal. The valence band spectra were typically recorded in between the first and last Pb *4f* measurements. Negligible changes are observed from the sequential measurements of core level spectra, indicating that the recorded valence band spectra presented in the following section are not substantially affected by beam-induced chemical changes.

Supplementary Discussion 1. **Potential influence of organic A-cation rotations and oscillations on the σ-π splitting and hot carrier cooling rate.**

The timescales of organic cation rotation/oscillation in MAPB and FAPB have been found, via IR spectroscopy, solid-state NMR, etc. to be in the range of 0.3 – 2 ps and 0.1 – 2 ps, respectively [28]. Our HERFD-XAS measurements were recorded with a 1 second integration/accumulation time per energy point, whereas the XAS process is ultrafast. Likewise, the density of states and XA spectrum simulations are based on instantaneous snapshots from ab initio molecular dynamics simulations. Consequently, we are sampling the time-averaged electronic structure over virtually instantaneous configurations, and find the σ-π splitting to be a time-averaged feature in the conduction band. This is analogous to sampling the time-averaged crystal structure of HaPs with bulk X-ray diffraction, which shows highly crystalline long-range order in spite of the picosecond-timescale structural disorder [29,30].

In the theoretical simulations, we find a positive correlation between the strength of N-H … Br hydrogen bonding and the time-averaged magnitude of the σ-π splitting: CsPB (no H-bonding, $\sigma\text{-}\pi_{calculated}$ = 3.5 eV) → MAPB (H-Br bond distance ~2.47 Å, $\sigma\text{-}\pi_{calculated}$ = 4.0 eV) → FAPB (H-Br bond distance ~ 2.37 Å, $\sigma\text{-}\pi_{calculated}$ = 4.2 eV). Thus, we suggest that the average strength of hydrogen-bonding strongly influences the average magnitude of the σ-π splitting. Organic A-cation rotation/oscillation rates may not strongly influence the magnitude of σ-π splitting. Methylammonium and formamidinium have comparable rotation/oscillation time constants and ionic radii (r_{MA+} = 2.70 Å, r_{FA+} = 2.79 Å), but FA^+ hydrogen-bonds more strongly to the halide framework [26,28]. Since the σ-π splitting is a persistent/time-averaged feature in the conduction band, it is expected to influence all electron dynamics (e.g. hot electron cooling rate, potential polaron formation), irrespective of their timescale(s).

Supplementary Discussion 2. **Potential connection between the σ-π character of the conduction band and polaron-like transport.**

Polaron formation, involving Coulomb screening of carriers by the Br-Pb sublattice against carrier-carrier, lattice defect and optical phonon scattering, has been invoked to explain the optoelectronic properties of HaPs[31]. We suggest that the σ states, having Br-Pb character, are delocalized/Bloch-like states while the π states, having Br-(A-cation) character, are spatially localized states. As femtosecond-timescale charge transfer between the organic A-cation and inorganic sublattice in the unoccupied states has been reported in MAPI (related compound), a timescale that could outcompete loss processes, we speculate that a non-thermalized electron undergoing transport will encounter both σ and π states and exhibit polaronic-like behavior (i.e. alternating between possibly lossless "trapping" and "detrapping")[32]. While occupying a localized π state, electrons may be "protected"/"screened" from scattering with electrons occupying σ states and lattice defects. The question of whether polaron dynamics can fully or partially explain the carrier dynamics of HaPs remains open[33].

Supplementary References

1. Stoumpos, C. C. *et al.* Crystal Growth of the Perovskite Semiconductor CsPbBr 3 : A New Material for High-Energy Radiation Detection. *Cryst. Growth Des.* **13**, 2722–2727 (2013).

2. Poglitsch, A. & Weber, D. Dynamic disorder in methylammoniumtrihalogenoplumbates (II) observed by millimeter-wave spectroscopy. *J. Chem. Phys.* **87**, 6373–6378 (1987).

3. Schueller, E. C. *et al.* Crystal Structure Evolution and Notable Thermal Expansion in Hybrid Perovskites Formamidinium Tin Iodide and Formamidinium Lead Bromide. *Inorg. Chem.* **57**, 695–701 (2018).

4. Trzhaskovskaya, M. B., Nefedov, V. I. & Yarzhemsky, V. G. Photoelectron Angular Distribution Parameters for Elements Z = 1 to Z = 54 in the Photoelectron Energy Range 100-5000 eV. *At. Data Nucl. Data Tables* **77**, 97–159 (2001).

5. Trzhaskovskaya, M. B., Nefedov, V. I. & Yarzhemsky, V. G. Photoelectron Angular Distribution Parameters for Elements Z = 55 to Z = 100 in the Photoelectron Energy Range 100-5000 eV. *At. Data Nucl. Data Tables* **82**, 257–311 (2002).

6. Drisdell, W. S. *et al.* Determining Atomic-Scale Structure and Composition of Organo-Lead Halide Perovskites by Combining High-Resolution X-ray Absorption Spectroscopy and First-Principles Calculations. *ACS Energy Lett.* **2**, 1183–1189 (2017).

7. Swarbrick, J. C., Skyllberg, U., Karlsson, T. & Glatzel, P. High Energy Resolution X-ray Absorption Spectroscopy of Environmentally Relevant Lead(II) Compounds. *Inorg. Chem.* **48**, 10748–10756 (2009).

8. Ekimova, M. *et al.* Aqueous Solvation of Ammonia and Ammonium: Probing Hydrogen Bond Motifs with FT-IR and Soft X-ray Spectroscopy. *J. Am. Chem. Soc.* **139**, 12773–12783 (2017).

9. Hou, Z. *et al.* Electronic structure of N-doped graphene with native point defects. *Phys. Rev. B* **87**, 165401 (2013).

10. Dalpian, G. M., Liu, Q., Varignon, J., Bibes, M. & Zunger, A. Bond disproportionation, charge self-regulation, and ligand holes in s- p and in d-electron ABX3 perovskites by density functional theory. *Phys. Rev. B* **98**, 075135 (2018).

11. Endres, J. *et al.* Valence and Conduction Band Densities of States of Metal Halide Perovskites: A Combined Experimental–Theoretical Study. *J. Phys. Chem. Lett.* **7**, 2722–2729 (2016).

12. Yang, J. *et al.* Accessing the Conduction Band Dispersion in CH 3 NH 3 PbI 3 Single Crystals. *J. Phys. Chem. Lett.* **12**, 3773–3778 (2021).

13. Mosconi, E., Amat, A., Nazeeruddin, M. K., Grätzel, M. & De Angelis, F. First-Principles Modeling of Mixed Halide Organometal Perovskites for Photovoltaic Applications. *J. Phys. Chem. C* **117**, 13902–13913 (2013).

14. Das, T., Di Liberto, G. & Pacchioni, G. Density Functional Theory Estimate of Halide Perovskite Band Gap: When Spin Orbit Coupling Helps. *J. Phys. Chem. C* **126**, 2184–2198 (2022).

15. Groot, F. de & Kotani, A. *Core Level Spectroscopy of Solids*. *Core Level Spectroscopy of Solids* (CRC Press, 2008).

16. Iihara, J., Fukuhara, K., Yagi, M., Omori, T. & Yoshihara, K. Chemical effects of L X-rays emitted from iodine and antimony compounds. *Hyperfine Interact.* **84**, 433–437 (1994).

17. Petric, M. & Kavčič, M. Chemical speciation via X-ray emission spectroscopy in the tender X-ray range. *J. Anal. At. Spectrom.* **31**, 450–457 (2016).

18. Aebli, M. *et al.* Lead-Halide Scalar Couplings in 207Pb NMR of APbX3 Perovskites (A = Cs, Methylammonium, Formamidinium; X = Cl, Br, I). *Sci. Rep.* **10**, 8229 (2020).

19. Rakita, Y., Kirchartz, T., Hodes, G. & Cahen, D. Type and Degree of Covalence: Empirical Derivation and Implications. *arXiv* (2019).

20. Sendner, M. *et al.* Optical Phonons in Methylammonium Lead Halide Perovskites and Implications for Charge Transport. *Mater. Horiz.* **3**, 613–620 (2016).

21. Miyata, K. *et al.* Large polarons in lead halide perovskites. *Sci. Adv.* **3**, e1701217 (2017).

22. Niesner, D. *et al.* Giant Rashba Splitting in CH3NH3PbBr3 Organic-Inorganic Perovskite. *Phys. Rev. Lett.* **117**, 126401 (2016).

23. Goldschmidt, V. M. Die Gesetze der Krystallochemie. *Naturwissenschaften* **14**, 477–485 (1926).

24. Travis, W., Glover, E. N. K., Bronstein, H., Scanlon, D. O. & Palgrave, R. G. On the application of the tolerance factor to inorganic and hybrid halide perovskites: a revised system. *Chem. Sci.* **7**, 4548–4556 (2016).

25. Shannon, R. D. Revised effective ionic radii and systematic studies of interatomic distances in halides and chalcogenides. *Acta Crystallogr. Sect. A* **32**, 751–767 (1976).

26. Amat, A. *et al.* Cation-Induced Band-Gap Tuning in Organohalide Perovskites: Interplay of Spin–Orbit Coupling and Octahedra Tilting. *Nano Lett.* **14**, 3608–3616 (2014).

27. Tao, S. *et al.* Absolute energy level positions in tin- and lead-based halide perovskites. *Nat. Commun.* **10**, 2560 (2019).

28. Gallop, N. P. *et al.* Rotational Cation Dynamics in Metal Halide Perovskites: Effect on Phonons and Material Properties. *J. Phys. Chem. Lett.* **9**, 5987–5997 (2018).

29. Yaffe, O. *et al.* Local Polar Fluctuations in Lead Halide Perovskite Crystals. *Phys. Rev. Lett.* **118**, 136001 (2017).

30. Singh, H. *et al.* Origin of the anomalous Pb-Br bond dynamics in formamidinium lead bromide perovskites. *Phys. Rev. B* **101**, 054302 (2020).

31. Zhu, X.-Y. & Podzorov, V. Charge Carriers in Hybrid Organic–Inorganic Lead Halide Perovskites Might Be Protected as Large Polarons. *J. Phys. Chem. Lett.* **6**, 4758–4761 (2015).

32. Man, G. J. *et al.* Electronic coupling between the unoccupied states of the organic and inorganic sublattices of methylammonium lead iodide: A hybrid organic-inorganic perovskite single crystal. *Phys. Rev. B* **104**, L041302 (2021).

33. Irvine, L. A. D., Walker, A. B. & Wolf, M. J. Quantifying polaronic effects on the scattering and mobility of charge carriers in lead halide perovskites. *Phys. Rev. B* **103**, L220305 (2021).
